# Acute Cervical Lymphadenitis Caused by *Mycobacterium florentinum*

**DOI:** 10.3201/eid1609.100433

**Published:** 2010-09

**Authors:** Salma S. Syed, Omolara Aderinboye, Kimberly E. Hanson, Eric D. Spitzer

**Affiliations:** Author affiliations: State University of New York, Stony Brook, New York, USA (S.S. Syed, O. Aderinboye, E.D. Spitzer);; University of Utah School of Medicine, Salt Lake City, Utah, USA (K.E. Hanson);; Associated Regional and University Pathologists Laboratories, Salt Lake City (K.E. Hanson)

**Keywords:** Bacteria, Mycobacterium florentinum, mycobacterial infections, atypical mycobacteria, acute cervical lymphadenitis, pediatrics, children, tuberculosis and other mycobacteria, letter

**To the Editor:** The incidence of nontuberculous mycobacterium (NTM) lymphadenitis appears to be rising, likely because of improved diagnostic techniques. *Mycobacterium avium-intracellulare* is the most common etiologic agent of NTM lymphadenitis in children (*1**,**2*). Newer diagnostic methods, including DNA sequencing, have identified cervical adenitis secondary to other slow-growing species (*M. lentiflavum*, *M. interjectum,* and, most recently *M. florentinum* in 2005) (*3**–**6*). We report a case of acute cervical lymphadenitis caused by *M. florentinum* in a child and briefly describe 4 other patients, both children and adults, with positive culture growth. These results suggest that *M. florentinum* infection is more widespread than previously appreciated.

A previously healthy girl, 3 years of age, came to our outpatient clinic with 2 months of bilateral cervical lymph node enlargement preceded by low-grade fevers for a few days. She had previously received clindamycin for 10 day without improvement. A tuberculin skin test showed 10 mm of induration, and results of a chest radiograph were negative. Both parents were from El Salvador, but the child was born in the United States and had never traveled abroad.

Her examination showed enlarged left (6 × 4 cm) and right (4 × 3 cm) posterior, cervical lymph nodes, which were indurated, erythematous, and fluctuant. Computed tomography scan of the neck showed multiple, bilateral, necrotic lymph nodes in the posterior cervical triangle, more notable on the right, with retropharyngeal abscesses (Figure).

**Figure:**
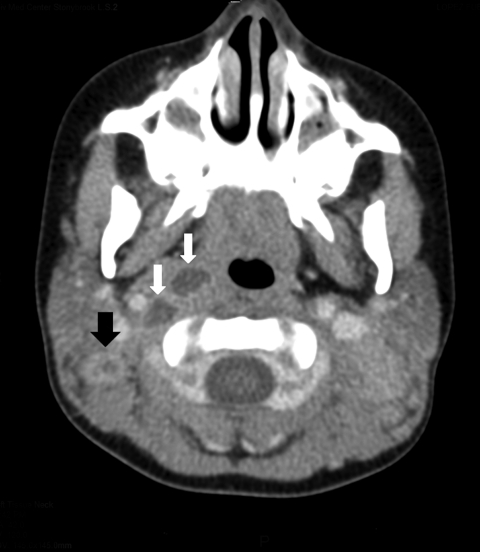
Computed tomography scan of the neck of a 3-year-old girl, showing right lateral retropharyngeal abscess (white arrows) and enlarged bilateral posterior cervical lymph nodes with low attenuation of a right cervical lymph node (black arrow), consistent with atypical mycobacterium adenitis.

The patient was admitted to the hospital because of concerns about airway obstruction. Laboratory findings included leukocyte count of 9.8 × 10^3^ cells/µL (40% neutrophils), increased thrombocytes (523 × 10^3^ cells/µL), and a routine, negative blood culture for bacterial growth. She had elevated alanine aminotransferase (822 U/L), aspartate aminotransferase (482 U/L), and lactate dehydrogenase (387 U/L) levels. Computed tomography scan of the abdomen showed no abscesses. She was given intravenous vancomycin.

The following day she underwent neck exploration, excisional left lymph node biopsy, and drainage of her retropharynx. Retropharyngeal cultures grew methicillin-resistant *Staphylococcus epidermidis* and *Streptococcus mitis*. Lymph node histopathologic analysis showed noncaseating granulomas with no malignancy. Results of tests with special stains for acid-fast bacilli and fungi were negative. She was discharged home after receiving oral linezolid for 10 days to complete 14 days of antimicrobial drug therapy. Her liver function test results had improved at the time of discharge.

The excised lymph node and neck abscess grew atypical mycobacteria, which were initially isolated by using an automated broth culture system (MGIT; Becton Dickinson, Sparks, MD, USA) after 4 weeks of incubation. Cultures were negative for *M. avium* complex by DNA hybridization, nonpigmented, and positive for nitrate reductase and inactivation of catalase at 68°C.

At the time of follow-up, the patient’s neck swelling had only slightly decreased, which resulted in complete surgical excision of bilaterally infected lymph nodes 3 weeks later with subsequent improvement. Repeat mycobacterial cultures were negative. No relapse occurred within 12 months of observation following surgery.

The isolate was identified as *M. florentinum* at Associated Regional and University Pathologists Laboratories (Salt Lake City, UT, USA) on the basis of sequencing of the first 500 bp of the 16S rRNA gene. The patient isolate exhibited 484/484 nt identities with the type strain of *M. florentinum*. The isolate was susceptible to amikacin and clarithromycin with MICs of 0.5 µg/mL but was resistant to ciprofloxacin (MIC 8 µg/mL), according to Clinical and Laboratory Standards Institute guidelines (www.clsi.org). Other drugs tested (with corresponding MICs) included ethambutol (4 µg/mL), gatifloxacin (2 µg/mL), moxifloxacin (1 µg/mL), rifampin (0.12 µg/mL), and streptomycin (0.5 µg/mL). No current Clinical and Laboratory Standards Institute interpretations are available for these agents.

Four additional cases of *M. florentinum* infection have been identified at Associated Regional and University Pathologists Laboratories since 2006 (after Institutional Review Board approval): a 76-year-old man (bronchial aspirate), a 47-year-old man (sputum), a 5-year-old girl (neck lymph node), and a 46-year-old man (unspecified source). Two of these cases were submitted from New York, 1 from Oregon, and 1 from Virginia.

*M. florentinum* is a recently described, slow-growing, nonpigmented mycobacterium. In 2005, Tortoli et al. described 8 strains obtained over 11 years from the sputum of patients with various pulmonary disorders and, in 1 case, from the lymph node of a 6-year-old girl (*6*). Seven of the 8 patients were hospitalized in Italy.

Atypical mycobacterium lymphadenitis generally shows unilateral lymph node involvement; however, the patient reported here had bilateral lymphadenitis complicated by retropharyngeal abscesses. Although disseminated NTM infection can lead to liver abnormalities, this patient had no risk factors for dissemination such as immunosuppression. Recommended treatment of atypical NTM lymphadenitis in children is complete surgical excision (*5**,**7*), although some studies have suggested antimicrobial drugs (*1**,**7**,**8*) or observation alone (*9*). *M. florentinum* exhibits resistance to several antimycobacterial drugs (*6*); therefore, surgical excision may still be the preferred treatment for this infection. Because the patient reported here had complete surgical excision and no recurrence, she did not require further antimicrobial drugs.

Cervical lymphadenitis in children is thought to result from ingestion of environmental mycobacteria (*10*). Although many NTM species are specific to particular geographic locations, our data suggest that *M. florentinum* is found in diverse locations in the United States. This organism appears to have a predilection for lymph nodes and lung tissue, similar to other NTM species.
